# Prenatal Ambient Air Pollution, Placental Mitochondrial DNA Content, and Birth Weight in the INMA (Spain) and ENVIR*ON*AGE (Belgium) Birth Cohorts

**DOI:** 10.1289/ehp.1408981

**Published:** 2015-08-28

**Authors:** Diana B.P. Clemente, Maribel Casas, Nadia Vilahur, Haizea Begiristain, Mariona Bustamante, Anne-Elie Carsin, Mariana F. Fernández, Frans Fierens, Wilfried Gyselaers, Carmen Iñiguez, Bram G. Janssen, Wouter Lefebvre, Sabrina Llop, Nicolás Olea, Marie Pedersen, Nicky Pieters, Loreto Santa Marina, Ana Souto, Adonina Tardón, Charlotte Vanpoucke, Martine Vrijheid, Jordi Sunyer, Tim S. Nawrot

**Affiliations:** 1 Center for Environmental Sciences, Hasselt University, Diepenbeek, Belgium; 2 Center for Research in Environmental Epidemiology (CREAL), Barcelona, Spain; 3 CIBER de Epidemiología y Salud Pública (CIBERESP), Institute of Health Carlos III, Madrid, Spain; 4 Institute for environmental medicine (IMM), Karolinska Institutet, Sweden; 5 Health Research Institute (BIODONOSTIA), Gipuzkoa, Spain; 6 Universitat Pompeu Fabra, Barcelona, Spain; 7 Center for Genomic Regulation (CRG), Barcelona, Spain; 8 Department of Radiology, University of Granada, Granada, Spain; 9 Instituto de Investigación Biosanitaria de Granada, ibs.GRANADA, Hospital Universitario San Cecilio, Granada, Spain; 10 Belgian Interregional Environment Agency, Brussels, Belgium; 11 Department of Obstetrics, East-Limburg Hospital, Genk, Belgium; 12 Foundation for the Promotion of Health and Biomedical Research in the Valencian Region (FISABIO), Valencia, Spain; 13 University of Valencia, Valencia, Spain; 14 Flemish Institute for Technological Research (VITO), Mol, Belgium; 15 INSERM (National Institute of Health and Medical Research), U823, Team of Environmental Epidemiology Applied to Reproduction and Respiratory Health, Institute Albert Bonniot, Grenoble, France; 16 Molecular Epidemiology of Cancer Unit, University Institute of Oncology, University of Oviedo, Oviedo, Spain; 17 IMIM (Hospital del Mar Research Institute), Barcelona, Spain; 18 Department of Public Health & Primary Care, Unit Environment & Health, Leuven University, Leuven, Belgium

## Abstract

**Background:**

Mitochondria are sensitive to environmental toxicants due to their lack of repair capacity. Changes in mitochondrial DNA (mtDNA) content may represent a biologically relevant intermediate outcome in mechanisms linking air pollution and fetal growth restriction.

**Objective:**

We investigated whether placental mtDNA content is a possible mediator of the association between prenatal nitrogen dioxide (NO2) exposure and birth weight.

**Methods:**

We used data from two independent European cohorts: INMA (n = 376; Spain) and ENVIRONAGE (n = 550; Belgium). Relative placental mtDNA content was determined as the ratio of two mitochondrial genes (MT-ND1 and MTF3212/R3319) to two control genes (RPLP0 and ACTB). Effect estimates for individual cohorts and the pooled data set were calculated using multiple linear regression and mixed models. We also performed a mediation analysis.

**Results:**

Pooled estimates indicated that a 10-μg/m3 increment in average NO2 exposure during pregnancy was associated with a 4.9% decrease in placental mtDNA content (95% CI: –9.3, –0.3%) and a 48-g decrease (95% CI: –87, –9 g) in birth weight. However, the association with birth weight was significant for INMA (–66 g; 95% CI: –111, –23 g) but not for ENVIRONAGE (–20 g; 95% CI: –101, 62 g). Placental mtDNA content was associated with significantly higher mean birth weight (pooled analysis, interquartile range increase: 140 g; 95% CI: 43, 237 g). Mediation analysis estimates, which were derived for the INMA cohort only, suggested that 10% (95% CI: 6.6, 13.0 g) of the association between prenatal NO2 and birth weight was mediated by changes in placental mtDNA content.

**Conclusion:**

Our results suggest that mtDNA content can be one of the potential mediators of the association between prenatal air pollution exposure and birth weight.

**Citation:**

Clemente DB, Casas M, Vilahur N, Begiristain H, Bustamante M, Carsin AE, Fernández MF, Fierens F, Gyselaers W, Iñiguez C, Janssen BG, Lefebvre W, Llop S, Olea N, Pedersen M, Pieters N, Santa Marina L, Souto A, Tardón A, Vanpoucke C, Vrijheid M, Sunyer J, Nawrot TS. 2016. Prenatal ambient air pollution, placental mitochondrial DNA content, and birth weight in the INMA (Spain) and ENVIRONAGE (Belgium) birth cohorts. Environ Health Perspect 124:659–665; http://dx.doi.org/10.1289/ehp.1408981

## Introduction

In recent years, traffic-related air pollution has been considered an important risk factor for adverse reproductive health effects. Prenatal exposure to nitrogen dioxide (NO_2_) has been associated with low birth weight, intrauterine growth restriction, and preterm birth (Pedersen et al. 2013; Stieb et al. 2012). Infants with low birth weight are at higher risk of mortality and morbidity and impaired cognitive development compared with infants with higher birth weight (Gluckman et al. 2008; Risnes et al. 2011).

Mitochondria are intracellular organelles that are essential for the aerobic production of adenosine triphosphate (ATP) by oxidative phosphorylation (OXPHOS). These “power plants of the cell” also play a critical role in signaling transduction for cell proliferation, apoptosis, calcium storage, and metabolism (Clay Montier et al. 2009; Lamson and Plaza 2002; Lee and Wei 2005). Several studies have identified the generation of oxidative stress, by producing reactive oxygen species (ROS), as one of the major mechanisms by which air pollution exerts adverse biological effects (Chahine et al. 2007; Li et al. 2008). Mitochondria are the major intracellular sources of ROS, which are generated under normal conditions as by-product of OXPHOS (Hou et al. 2010). On the other hand, mitochondria are also the primary targets of oxidative stress because, in comparison with nuclear DNA (nDNA), mitochondrial DNA (mtDNA) lacks the protective strategies associated with nDNA, such as protective histones, chromatine structure, and sufficient DNA repair capacity (Lee and Wei 2000). Consequently, mtDNA is particularly vulnerable to ROS-induced damage and has a high mutation rate (Lamson and Plaza 2002). Mitochondria compensate for these mutations by increasing their number and their replication rate, resulting in a change in mtDNA content, which therefore reflects mitochondrial damage and dysfunction (Clay Montier et al. 2009; Hou et al. 2010; Lamson and Plaza 2002; Sahin et al. 2011).

The placenta plays a unique role in the transfer of gases, nutrients, and waste between the mother and developing child. It is both a metabolic and an endocrine organ. However, the placenta has a limited capability to metabolize a large number of foreign compounds (Storvik et al. 2014). The placenta requires energy to maintain its function, and this energy provision is regulated by mitochondrial function of placenta cells (Myllynen et al. 2005). Air pollution exposure is hypothesized to affect the fetus directly through transplacental exposure or indirectly by affecting maternal health and body functions (Morello-Frosch et al. 2010). This could impair the placental exchange of nutrients and gases. Under poor nutritional conditions the fetus can adapt its mitochondrial structure and metabolism. Therefore, this “metabolic reprogramming” could be at the origin of adverse birth outcomes (Gemma et al. 2006).

Recently, it has been shown that exposure to particulate air pollution during pregnancy was associated with placental mtDNA content (Janssen et al. 2012). We hypothesized that changes in mtDNA content may represent a biological causal effect along the path linking air pollution exposure with the potential adverse health effects of the offspring. Given two independent European birth cohorts (INMA and ENVIR*ON*AGE), we aimed to assess the role of mediating effects of placental mtDNA content on the association of prenatal NO_2_ exposure with birth weight.

## Materials and Methods

*Study design and population.* The Spanish population-based birth cohort study INMA (INfancia y Medio Ambiente; Childhood and Environment) recruited pregnant women in four centers (Valencia, Sabadell, Gipuzkoa, and Asturias), following a common protocol (Guxens et al. 2012). A total of 2,616 pregnant women were enrolled between 2004 and 2008 during the first trimester of pregnancy at public primary health care centers or public hospitals if they fulfilled the inclusion criteria: ≥ 16 years of age, a singleton pregnancy, intention to deliver at the reference hospital, no problems of communication, and no assisted conception. Of all eligible pregnant women, 57% agreed to participate. The present analysis included 390 mother–newborn pairs from the INMA cohort with placental mtDNA content data. A comparison of this INMA subcohort with the whole INMA cohort (*n* = 2,616) did not show differences in maternal age, pregestational body mass index (BMI), parity, and ethnicity.

In the population-based birth cohort study ENVIR*ON*AGE (ENVIRonmental influence ON AGEing), 556 pregnant women were enrolled between 2010 and 2013 at the South-East-Limburg Hospital in Genk (Belgium) when they arrived for delivery. The inclusion criteria were ≥ 18 years of age, singleton pregnancy, and ability to fill out questionnaires in Dutch. The overall participation rate of eligible mothers was 56%. A comparison of the ENVIR*ON*AGE birth cohort with all births in Flanders (Cox et al. 2013) did not show differences in maternal age, pregestational BMI, parity, and ethnicity.

Study approval was obtained from the ethics committees of each participating center and written informed consent was obtained from the mothers. In both cohorts information on maternal age, ethnicity, maternal smoking status, place of residence, prepregnancy BMI, and parity was obtained. In INMA they were obtained by questionnaires and interviews, in ENVIR*ON*AGE by questionnaires. Perinatal parameters such as newborn’s sex, birth date, birth weight, gestational age, and delivery by cesarean section were collected by birth records. Details about maternal and child characteristics were standardized to perform a harmonized, pooled analysis.

*Sample collection.* In the INMA cohort, placentas were randomly collected in approximately one of four deliveries (*n* = 502). The entire placentas were frozen after delivery at –20°C until they were transferred on dry ice to the Hospital Universitario San Cecilio (HUSC) Biobank in Granada (Spain) and stored at –86°C. No information was available about the time between delivery and storage at the different Spanish hospitals involved in the study. MtDNA content was measured in 390 out of the 502 randomly selected placentas in INMA. In the ENVIR*ON*AGE cohort, all placentas (*n* = 556) were collected after delivery and were frozen within 10 min at –20°C. In both cohorts, placentas were thawed minimally to obtain tissue biopsies for DNA extraction. To minimize the impact of within-placenta variability, biopsies were all taken 1–1.5 cm below the chorioamniotic membrane at a fixed location and preserved at –80°C (Janssen et al. 2012).

*DNA extraction and measurement of mtDNA content.* In the INMA cohort, DNA was extracted from placental tissue cells using the DNeasy Blood & Tissue Kit (Qiagen, Valencia, CA, USA) following the manufacturer’s instructions. In the ENVIR*ON*AGE cohort, DNA was extracted from placental tissue cells using the QIAamp DNA minikit (Qiagen Inc., Venlo, Netherlands) following the manufacturer’s instructions. In both cohorts, DNA samples were quantified using the Nanodrop spectrophotometer (ND-1000; Isogen Life Science, De Meern, Netherlands) and the Quant-iT™ PicoGreen® dsDNA Assay Kit (Life Technologies, Foster City, CA, USA) using the Omega Fluostar plate reader (BMG LABTECH, Ortenberg, Germany).

MtDNA content was measured in placental tissue cells by determining the ratio of two mitochondrial genes [mitochondrial encoded NADH dehydrogenase subunit 1 (*MT-ND1*) and mitochondrial forward primer for nucleotide 3212 and reverse primer from nucleotide 3319 (*MTF3212/R3319*)] to two nuclear control genes [acidic ribosomal phosphoprotein P0 (*RPLP0*), and beta-actin (*ACTB*)] using a quantitative real-time polymerase chain reaction (qPCR) assay (Janssen et al. 2012). qPCR was performed using 2.5 μL of extracted DNA (5 ng/μL) and 7.5 μL of master mix consisting of Fast SYBR® Green I dye 2× (5 μL/reaction; Life Technologies), forward (0.3 μL/reaction–300 nM) and reverse (0.3 μL/reaction–300 nM) primer (Biolegio, Nijmegen, Netherlands) and RNase free water (1.9 μL/reaction). Samples were run in triplicate in a 384-well format. qPCR was performed using the 7900HT Fast Real-Time PCR System (Life Technologies) with the following thermal cycling profile: 20 sec at 95°C (activation), followed by 40 cycles of 1 sec at 95°C (denaturation) and 20 sec at 60°C (annealing/extension). At the end of each run, a melting curve analysis was performed to confirm amplification specificity and absence of primer dimers (15 sec at 19°C, 15 sec at 60°C, 15 sec at 95°C). qBase software (Biogazelle, Zwijnaarde, Belgium) was used to normalize data and correct for run-to-run differences (Hellemans et al. 2007).

*Ambient air pollution assessment.* In INMA, ambient concentrations of NO_2_ were measured with the aid of passive samplers (Radiello, Fundazione Salvatore Maugeri, Padua, Italy) distributed outside across the study areas according to geographic criteria, taking into account the expected pollution gradients and the distribution of the residences of the participating women. The samplers remained exposed during various 7-day sampling periods through pregnancy. The methodology has been described in detail elsewhere (Aguilera et al. 2008; Iñiguez et al. 2009), and further sampling information is given in Supplemental Material, Table S1. Temporally adjusted land use regression (LUR) models were used to predict NO_2_ levels at women’s residential addresses and taking into account residential changes if women lived at least 2 months of pregnancy in the new residence. To calculate individual exposure during pregnancy, annual average NO_2_ estimates from the LUR were temporally adjusted using serial records from the network of monitoring stations covering each of the four INMA study areas. The validation statistics gave a spatial explained variance (*R* ^2^) for annual mean NO_2_ from 0.52 to 0.75 in the four INMA subcohorts (see Supplemental Material, Table S1).

In ENVIR*ON*AGE, regional background levels of NO_2_ for each woman’s home address were calculated using a kriging interpolation method (Jacobs et al. 2010; Janssen et al. 2008) that uses land cover data obtained from satellite images combined with a dispersion model (Lefebvre et al. 2011). This model chain provided NO_2_ values, combining data from the Belgian telemetric air quality network, point sources and line sources, which were then interpolated to a high-resolution receptor grid. This method was used to obtain NO_2_ levels at women’s residential addresses, taking into account any residential change during pregnancy. The validation statistics gave a temporal explained variance (*R* ^2^) for hourly averages > 0.80 and a spatial explained variance (*R* ^2^) for annual mean NO_2_ of 0.82.

To explore potentially critical exposures during pregnancy, we calculated individual NO_2_ concentrations for the three trimesters of pregnancy using the same procedure used for the entire pregnancy: 1–13 weeks starting from date of conception (trimester 1), 14–28 weeks (trimester 2), and 29 weeks to delivery (trimester 3).

More details on exposure measurements for INMA and ENVIR*ON*AGE can be found in Supplemental Material, Table S1.

*Statistical analysis.* Continuous data were checked for normality using the Shapiro–Wilk test statistic. Placental mtDNA content data were right skewed and therefore logarithmically transformed (log_10_). Generalized additive models (GAMs) were used to assess the linearity of the associations between *a*) prenatal NO_2_ exposure and mtDNA content, *b*) mtDNA content and birth weight, and *c*) prenatal NO_2_ exposure and birth weight (see Supplemental Material, Figure S1). Multiple linear regression models were used in ENVIR*ON*AGE. Multiple linear mixed models with a random cohort effect were used in INMA and in the pooled data set (four INMA cohorts and ENVIR*ON*AGE). Covariates used in the model were gestational age (linear and quadratic term), newborn’s sex, maternal age, ethnicity, parity, smoking status, education, season of birth (January–March, April–June, July–September, October–December), and prepregnancy maternal BMI. For the present analysis, we excluded 14 mother–newborn pairs from INMA and 6 mother–newborn pairs from ENVIR*ON*AGE with missing values in the outcome, exposure, and confounders. After these exclusions, the final study population consisted of 376 subjects for INMA and 550 subjects for ENVIR*ON*AGE.

To determine whether placental mtDNA content is a potential mediator of the association between prenatal NO_2_ exposure and birth weight, we performed a mediation analysis. The direct effect (DE), indirect effect (IE), and total effect (TE) were estimated using the SAS macro developed by Valeri and VanderWeele (2013). When assumptions of the mediation analysis hold, the DE represents the effect of prenatal NO_2_ exposure on birth weight after controlling for mtDNA content, and the IE is the estimated effect of NO_2_ exposure during pregnancy operating through mtDNA content (Valeri and VanderWeele 2013). The proportion of mediation by placental mtDNA content was calculated as the ratio of IE to TE.

A sensitivity analysis was performed, in which all nonvaginal deliveries were excluded, because it has been suggested that the fetus could be exposed to different levels of oxidative stress depending on the type of delivery (Inanc et al. 2005). In another sensitivity analysis we used cohort as a fixed effect instead of a random effect. Further, we tested the interaction between mtDNA content and sex on birth weight by including its interaction term in the full model. In INMA we also performed an additional sensitivity analysis taking into account the time–activity patterns of the women during pregnancy. Because it has been indicated that time–activity patterns during pregnancy should be considered to improve the accuracy of exposure measurement and reduce exposure misclassification (Estarlich et al. 2011), we calculated the time spent at home from self-reported information (questionnaire at week 32) and restricted in INMA our analysis to women who spent ≥ 15 hr/day at home (Estarlich et al. 2011). This information was not available for the ENVIR*ON*AGE cohort.

All statistical analysis were conducted using SAS software (version 9.3; SAS Institute Inc., Cary, NC, USA).

## Results

*Characteristics of the study population.* Characteristics of the 376 and 550 mother–newborn pairs in, respectively, the INMA and the ENVIR*ON*AGE cohort are shown in Table 1. INMA mothers were more likely to be primiparous, older, lower educated, of European origin, and had lower BMI compared with mothers from ENVIR*ON*AGE. The mean birth weight was lower and placental mtDNA content higher in INMA newborns compared with newborns from ENVIR*ON*AGE.

**Table 1:** Characteristics of INMA and ENVIR*ON*AGE participants.

Characteristics	INMA (*n *= 376)	ENVIR*ON*AGE (*n *= 550)
Maternal
Age (years)	32.2 ± 3.9*	29.0 ± 4.6*
Smoking
Never	170 (45.2)*	354 (64.4)*
Quit smoking before week 12	143 (38.0)*	119 (21.6)*
During entire pregnancy	63 (16.8)*	77 (14.0)*
Education
Primary school or none	75 (20.0)*	67 (12.2)*
Secondary school	167 (44.4)*	203 (36.9)*
University	134 (35.6)*	280 (50.9)*
Parity
1	212 (56.4)	299 (52.4)
2	138 (36.7)	195 (35.5)
≥ 3	26 (6.9)	56 (10.2)
Prepregnancy BMI (kg/m^2^)	23.5 ± 4.4	24.1 ± 4.5
Ethnicity
European	343 (91.2)	485 (88.2)
Non-European	33 (8.8)	65 (11.8)
Cohort
Valencia	63 (16.8)	NA
Asturias	37 (9.8)	NA
Sabadell	120 (31.9)	NA
Gipuzkoa	156 (41.5)	NA
ENVIR*ON*AGE	NA	550 (100.0)
Time spent at home
> 15 hr/day	214 (56.9)	NA
≤ 15 hr/day	162 (43.1)	NA
Newborn
Gestational age (weeks)	39.9 ± 1.3	39.3 ± 1.2
Sex
Male	194 (51.6)	277 (50.4)
Female	182 (48.4)	273 (49.4)
Season at birth
January–March	99 (26.3)	156 (28.4)
April–June	102 (27.1)	131 (23.8)
July–September	92 (24.5)	143 (26.0)
October–December	83 (22.1)	120 (21.8)
Preterm delivery (< 37 weeks)
Yes	7 (1.9)	14 (2.6)
No	369 (98.1)	536 (97.5)
Vaginal delivery
No	322 (85.6)	521 (94.7)
Yes	54 (14.4)	29 (5.3)
Birth weight (g)	3,290 ± 423*	3,429.6 ± 432*
Placental mtDNA content	1.3 (1.1–1.5)*	1.0 (0.7–1.4)*
NA, not applicable. Continuous covariates expressed by mean ± SD or geometric mean and 25–75th percentile; categorical covariates are described by frequencies (%). Differences between cohorts were assessed using independent *t*-tests. Subjects without available information have been excluded before performing the independent *t*-tests.*p < 0.05.		

The mean (± SD) pregnancy average exposure to NO_2_ was 25.5 ± 11.4 μg/m^3^ and 21.1 ± 4.2 μg/m^3^ in INMA and ENVIR*ON*AGE, respectively. Similar differences in exposure levels between cohorts were observed in the mean trimester concentrations (Table 2).

**Table 2:** Descriptive statistics of prenatal NO_2_ exposure (μg/m^3^).

NO_2_ exposure (μg/m^3^)	Mean ± SD	P5	P25	P50	P75	P95	*r*^*a*^
INMA (*n *= 376)
Trimester 1	26.1 ± 12.9	5.6	16.4	23.1	33.7	74.2	0.91*
Trimester 2	25.6 ± 11.6	5.7	16.4	24.8	31.2	74.7	0.93*
Trimester 3	25.7 ± 12.1	5.7	16.9	23.8	32.3	74.4	0.92*
Entire pregnancy	25.5 ± 11.4	5.7	17.2	24.0	32.3	66.7	—
ENVIR*ON*AGE (*n *= 550)
Trimester 1	20.7 ± 6.1	7.3	16.3	20.2	24.9	39.2	0.61*
Trimester 2	20.8 ± 6.0	8.6	16.2	20.5	25.1	46.0	0.86*
Trimester 3	21.4 ± 6.1	9.2	16.9	20.8	25.6	40.3	0.66*
Entire pregnancy	21.1 ± 4.2	12.6	18.2	20.8	23.7	40.3	—
INMA + ENVIR*ON*AGE (*n *= 926)
Trimester 1	22.7 ± 9.8	5.6	16.1	21.2	26.8	74.2	0.86*
Trimester 2	22.6 ± 9.1	5.7	15.9	21.3	27.3	74.7	0.91*
Trimester 3	23.0 ± 9.3	5.7	16.7	21.5	27.3	74.4	0.88*
Entire pregnancy	22.7 ± 8.3	5.7	17.6	21.2	25.6	66.7	—
P, percentile. Continuous covariates expressed by mean ± SD.^***a***^Pearson correlation between the pregnancy average and trimester-specific exposures. *p < 0.001.							

*Association between placental mtDNA content and NO_2_ exposure.* In the INMA cohort, NO_2_ exposure during each trimester and the entire pregnancy was negatively and significantly associated with placental mtDNA content (Table 3). These results were consistent in the direction of the associations in the four different INMA subcohorts (see Supplemental Material, Table S2). Each 10-μg/m^3^ increment in average pregnancy exposure was associated with a lower placental mtDNA content of 5.5% [95% confidence interval (CI): –8.8, –2.1%]. In the ENVIR*ON*AGE cohort, NO_2_ exposure was also negatively associated with placental mtDNA content in each trimester and in the entire pregnancy, but it was statistically significant only during the second (–11.1%; 95% CI: –19.9, –1.2%) and third trimesters of pregnancy (–13.5%; 95% CI: –20.1, –6.4%) (Table 3). The pooled analysis showed a statistically significant association with exposure during the second and third trimesters as well as in the entire pregnancy (Table 3).

**Table 3:** Percent change in placental mtDNA content in association with prenatal NO_2_ exposure in INMA, ENVIR*ON*AGE, and in the pooled sample.

Pregnancy period	Differences in placental mtDNA content (%) (95% CI)	*p*-Value
INMA (*n *= 376)^*a*^^,^^*b*^
Trimester 1	–4.1 (–7.1, –1.1)	0.007
Trimester 2	–5.0 (–8.0, –2.0)	0.002
Trimester 3	–4.9 (–7.9, –1.8)	0.003
Entire pregnancy	–5.5 (–8.8, –2.1)	0.002
ENVIR*ON*AGE (*n *= 550)
Trimester 1	–5.1 (–15.5, 6.6)	0.38
Trimester 2	–11.1 (–19.9, –1.24)	0.03
Trimester 3	–13.5 (–20.1, –6.4)	0.003
Entire pregnancy	–10.1 (–20.1, 1.24)	0.08
INMA + ENVIR*ON*AGE (*n *= 926)^*c*^
Trimester 1	–2.5 (–6.4, 1.6)	0.22
Trimester 2	–4.4 (–8.4, –0.3)	0.04
Trimester 3	–5.2 (–9.1, –1.2)	0.01
Entire pregnancy	–4.9 (–9.3, –0.3)	0.04
Effect size was estimated for each 10-μg/m^3^ increment in exposure to NO_2_ at each mother’s residence during the corresponding period. Models were adjusted for newborn’s sex, maternal age, maternal smoking status, gestational age (linear and quadratic), prepregnancy BMI, parity, ethnicity, season of birth, and education.^***a***^Results followed the same direction in all four INMA subcohorts (see Supplemental Material, Table S2). ^***b***^Four INMA subcohorts were included as random effect. ^***c***^Cohorts were included as random effect.		

*Association between birth weight and NO_2_ exposure.* The association between birth weight and prenatal NO_2_ exposure was significant in the INMA cohort for all three trimesters of pregnancy (Table 4). Each 10-μg/m^3^ increment in average pregnancy NO_2_ exposure was associated with a 66.4 g (95% CI: –111.0, –22.7) decrease in birth weight (Table 4). These results were consistent in the direction of the associations in the four different INMA subcohorts (see Supplemental Material, Table S3). In the ENVIR*ON*AGE cohort, estimates were in the same direction although the estimated effects were smaller than in INMA and did not reach significance (–19.8 g; 95% CI: –101.1, 61.7). After both cohorts were pooled, the association between birth weight and NO_2_ was significant in all pregnancy trimesters and in the entire pregnancy (–47.5 g; 95% CI: –86.6, –8.5) (Table 4).

**Table 4:** Association between prenatal NO_2_ exposure and birth weight in INMA, ENVIR*ON*AGE, and in the pooled sample.

Pregnancy period	Differences in birth weight (g) (95% CI)	*p*-Value
INMA (*n *= 376)^*a*^^,^^*b*^
Trimester 1	–56.2 (–94.5, –17.8)	0.004
Trimester 2	–56.3 (–96.2, –16.4)	0.006
Trimester 3	–52.1 (–93.8, –12.5)	0.01
Entire pregnancy	–66.4 (–111.0, –22.7)	0.004
ENVIR*ON*AGE (*n *= 550)
Trimester 1	–20.0 (–91.3, 51.3)	0.58
Trimester 2	–3.4 (–76.4, 69.5)	0.93
Trimester 3	–29.9 (–98.2, 38.3)	0.39
Entire pregnancy	–19.8 (–101.1, 61.7)	0.63
INMA + ENVIR*ON*AGE (*n *= 926)^*c*^
Trimester 1	–44.1 (–77.4, –10.8)	0.01
Trimester 2	–36.2 (–70.9, –1.6)	0.04
Trimester 3	–37.5 (–71.4, –3.6)	0.03
Entire pregnancy	–47.5 (–86.6, –8.5)	0.02
Effect size was estimated for each 10-μg/m^3^ increment in exposure to NO_2_ at each mother’s residence during the corresponding period. Models were adjusted for newborn’s sex, season of birth, maternal age, maternal smoking status, parity, ethnicity, education, gestational age (linear and quadratic), and prepregnancy BMI.^***a***^Results followed the same direction in all four INMA subcohorts^.^ (see Supplemental Material, Table S3). ^***b***^Four INMA subcohorts were included as random effect. ^***c***^Cohorts were included as random effect.		

*Association between placental mtDNA content and birth weight.* Placental mtDNA content was positively and significantly associated with birth weight in both cohorts and in the pooled analysis (Table 5). Interaction tests showed that the interaction of mtDNA content with sex was significant for the individual cohorts and the pooled analysis. This suggests evidence of effect modification by sex. In the pooled analysis, an interquartile range (IQR) increase in mtDNA content was associated with a 66-g (95% CI: 18, 114) increase in mean birth weight in boys, compared with 26 g (95% CI: –67, 15) in girls (interaction *p-*value = 0.009) (Table 5).

**Table 5:** Association between placental mtDNA content and birth weight (g) in INMA, EVIR*ON*AGE, and in the pooled sample.

	INMA^*a*^^,^^*b*^				ENVIR*ON*AGE				INMA + ENVIR*ON*AGE^*c*^			
	*n*	Differences in birth weight (g) (95% CI)	*p*-Value	Interaction *p*-value	*n*	Differences in birth weight (g) (95% CI)	*p*-Value	Interaction *p*-value	*n*	Differences in birth weight (g) (95% CI)	*p*-Value	Interaction *p*-value
All	376	249.0 (83.6, 414.3)	0.003	0.003	550	129.2 (7.8, 259.0)	0.04	0.04	926	140.2 (43.2, 237.2)	0.005	0.009
Boys	194	124.0 (45.6, 202.5)	0.002	NA	277	34.0 (–34.4, 102.4)	0.33	NA	471	65.9 (17.9, 114.0)	0.007	NA
Girls	182	–2.44 (–80.5, 75.6)	0.95	NA	273	–15.2 (–69.3, 39.0)	0.58	NA	455	26.4 (–67.4, 14.6)	0.21	NA
NA, not applicable. Effect size was estimated for each IQR increment (INMA = 0.58; ENVIR*ON*AGE = 0.77; pooled sample = 0.76) in mtDNA content. ^***a***^Models were adjusted for gestational age (linear and quadratic), newborn’s sex, maternal age, maternal smoking status, prepregnancy BMI, parity, ethnicity, season of birth, education, and interaction term sex and mtDNA content. ^***b***^Four INMA subcohorts were included as random effect. ^***c***^Cohorts were included as random effect.												

**Figure 1:**
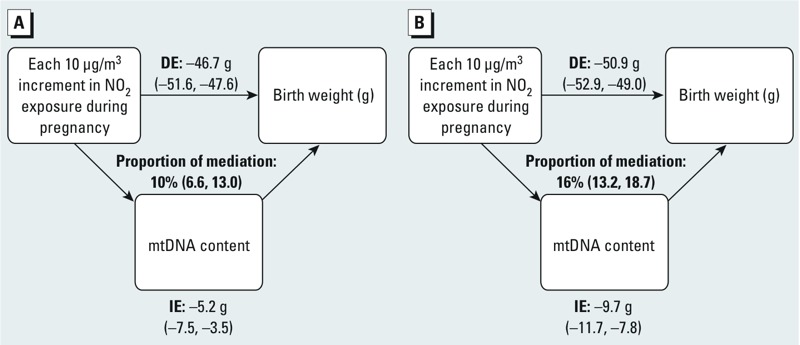
Mediation analysis of the estimated effect (95% CIs) of prenatal NO_2_ exposure (μg/m^3^) on birth weight through placental mtDNA content in the INMA cohort. (*A*) Whole INMA; (*B*) INMA boys. Results from mediation analysis with exposure to NO_2_ during the entire pregnancy were obtained using the SAS macro developed by Valeri and VanderWeele (2013). The figure shows placental mtDNA as a potential mediator, the estimates of indirect effect (IE), the estimates of the direct effect (DE), and proportion of mediation. Model was adjusted for gestational age (linear and quadratic term), newborn’s sex, maternal age, maternal smoking status, prepregnancy BMI, parity, ethnicity, season, education, and INMA subcohort.

*Mediation analysis.* In INMA, the mediation analysis suggested that 10% (95% CI: 6.6, 13.0) of the association between birth weight and pregnancy average NO_2_ exposure may be mediated by differences in placental mtDNA content (Figure 1A). The corresponding estimates for mediation of associations between birth weight and NO_2_ exposure during the first, second, and third trimesters, were 9.1% (95% CI: 5.3, 12.6), 11.4% (95% CI: 7.3, 15.2), and 12.2% (95% CI: 7.7, 16.3), respectively. When we limited the mediation analysis to boys in the INMA cohort, the analysis suggested a mediation effect of 16% (95% CI: 18.7, 13.2) (Figure 1B). Because in the ENVIR*ON*AGE cohort prenatal NO_2_ exposure was not significantly associated with birth weight, we did not perform the subsequent mediation analysis. After pooling both cohorts, the estimated proportion of mediation by mtDNA content was not significant for the association between birth weight and pregnancy average NO_2_ exposure (2.2%; 95% CI: –2.0, 6.1), or for NO_2_ exposure during the different trimesters (see Supplemental Material, Table S4). Mediation analysis of the pooled data for boys suggested placental mtDNA content mediated 6.4% (95% CI: 2.4, 10.0) of the association between NO_2_ during trimester 3 and birth weight, but mediation was not statistically significant for average pregnancy NO_2_ or NO_2_ during trimester 1 or 2 (see Supplemental Material, Table S4).

*Sensitivity analysis.* The reported associations did not change after excluding mother–newborn pairs with nonvaginal deliveries (*n* = 83) (data not shown). Furthermore, when we used cohort as a fixed effect, our reported estimates did not change either (data not shown). Finally, associations were stronger when the analysis was limited to 214 INMA participants who spent ≥ 15 hr/day at home, including associations between NO_2_ and birth weight (e.g., for average pregnancy NO_2_: 84 g; 95% CI: –142, –26 compared with –66 g; 95% CI: –111, –23) (see Supplemental Material, Tables S5 and S6, respectively).

## Discussion

Mitochondria, the energy producers of the cells, are particularly sensitive to environmental toxicants because they lack repair capacity (Clay Montier et al. 2009). Fetuses adapt their mitochondrial structure and metabolism when the supply of nutrients is limited (Gemma et al. 2006). We hypothesized that mitochondrial damage may be a causal intermediate in biological mechanisms linking air pollution to birth outcomes. In the present study, we evaluated placental mtDNA content, a proxy of mitochondrial damage, as a potential mediator of the association between reduced birth weight and prenatal NO_2_ exposure. The key findings, based on two independent European cohorts were that *a*) prenatal NO_2_ exposure is inversely associated with placental mtDNA content; *b*) placental mtDNA content is positively associated with birth weight; *c*) prenatal NO_2_ exposure is inversely associated with birth weight; and *d*) placental mtDNA content can be a potential mediator of the association between birth weight and prenatal NO_2_ exposure.

Ambient air contains a mixture of pollutants. NO_2_ is frequently used as a surrogate for traffic-related air pollution because it is considered to be a good proxy of other pollutants originating from the same sources (World Health Organization 2006). An appreciable number of epidemiologic studies have shown an association between fetal growth restriction and prenatal exposure to air pollution with evidence of statistical significant heterogeneity in the estimated effects among different locations (Parker et al. 2011; Stieb et al. 2012). An earlier observation on the full INMA cohort (*n* = 2,337) reported an estimated decrease in birth weight of 11 g for every 10-μg/m^3^ increment in NO_2_ (Estarlich et al. 2011). Data from 14 European mother–child cohorts, including INMA, reported a very weak association between birth weight of 1 g (95% CI: –6, 4 g) and NO_2_ during pregnancy (Pedersen et al. 2013). In our present study we estimated a 48-g reduction in birth weight (95% CI: –87, –9) with a 10-μg/m^3^ increment in pregnancy average NO_2_ based on the pooled analysis, with a stronger association in the INMA cohort (–66 g; 95% CI: –111, –23) than in the ENVIR*ON*AGE cohort (–20 g; 95% CI: –101, 62). This association also varied among the four INMA subcohorts, ranging from –12 g (95% CI: –20, 12; *n* = 63) for the Valencia subcohort to –156 g (95% CI: –378, 67; *n* = 37) for the Asturias subcohort (see Supplemental Material, Table S4). Differences in effect sizes across the studied cohorts might be attributable to different levels of exposure, population variability, and variation in meteorological conditions, but also noncausal mechanisms could explain these differences—for example, differences in study design and conduct, exposure assessment, and differences in residual confounding. Nevertheless, our GAM plots showed linear associations between NO_2_ and mtDNA content, NO_2_ and birth weight, and mtDNA content and birth weight in both cohorts (see Supplemental Material, Figure S1).

We need to consider that our exposure assessment was limited to the residential address of the mothers: Exposure to other air pollutants (e.g., particulate matter and carbon monoxide), environmental and dietary contaminants that have been associated with lower birth weight, as well as exposure to NO_2_ during a commute, at work, and elsewhere was not taken into account. Time spent at home can influence associations between ambient air pollution measured at the mother’s residence and birth weight (Estarlich et al. 2011); in this regard we also found stronger associations within INMA for mothers who spend > 15 hr/day at home.

In contrast to the epidemiological evidence, the mechanisms responsible for fetal growth restriction due to air pollution are largely unknown. Hypotheses are that air pollutants could cause oxidative stress or inflammation, alter placental growth, decrease placental exchange of nutrients and gases, foster endocrine disruption, or cause maternal health effects—all of which could possibly lead to altered fetal growth (Kannan et al. 2006). Oxidative stress–induced DNA damage appears to be a particularly important mechanism of action of urban air pollutants (Møller et al. 2014). MtDNA is particularly vulnerable to ROS-induced damage and has been described as a proxy of air pollution–induced damage (Byun and Baccarelli 2014; Hou et al. 2010). In this study, we estimated that 10% (95% CI: 6.6, 13.0%) of low birth weight caused by prenatal NO_2_ exposure could be explained by placental mtDNA content. This finding was demonstrated in a subsample of 376 mother–child pairs of the INMA cohort. We are aware that epidemiological studies can only show associations, but cannot prove causality; nevertheless, our formal mediation analysis is based on a predefined hypothesis and is in line with experimental evidence.

Mechanisms through which prenatal exposure to traffic-related air pollution might cause placental inflammation and oxidative stress are unclear. The maternal and fetal circulation are separated by the placental barrier that is formed by the syncytiotrophoblast layer, which faces the maternal environment (Wick et al. 2010). This barrier contains placental transporters that can block or facilitate foreign compounds (Myllynen et al. 2005; Wick et al. 2010). Further, it has been reported that air pollution was associated with increased white blood cells in chronic obstructive pulmonary disease (COPD) patients, suggesting that air pollutants can elicit systemic inflammation (Bose et al. 2015). In addition, it has been observed that human plasma collected from individuals exposed to diesel exhaust for only short periods of time (1 hr) is proinflammatory to endothelial cells *in vitro* (Channell et al. 2012), implying that soluble, proinflammatory mediators circulate in the blood after inhalation of diesel. From these studies it might be hypothesized that maternal circulating proinflammatory mediators are responsible for associations of prenatal NO_2_ with placental mtDNA content and birth weight in our study population. Other mechanisms might include transient receptor potential (TRP) channels which are highly expressed in placenta, and their activation has been suggested to play important roles in placental development and regulating the fetal–maternal interface in mice models (Weldy et al. 2014). If air pollution exposure can result in systemic activation of TRP channels, we might speculate that placental TRP channels are also activated and may mediate our observed effects. In this context, it has been shown recently that TRP channels interact with a large number of mitochondrial proteins (Feng et al. 2013).

Induction of ROS levels stimulates autophagy and mitophagy as exemplified by lower mtDNA content in placental tissue (Kubli and Gustafsson 2012). In the present study, stratified analyses indicated a stronger inverse association between placental mtDNA content and prenatal NO_2_ exposure in newborn boys than in girls. Indeed sex-dependent susceptibility to oxidative stress has been shown and the antioxidant defenses are apparently different in XX and XY cells (Malorni et al. 2008).

Our study has some limitations. Ambient exposure to air pollution does not account for indoor exposure, which has also been associated with reduced birth weight (Nieuwenhuijsen et al. 2013). Although our results were consistent after multiple adjustments, some residual confounding by some unknown factors that are associated with ambient air pollution, mitochondrial function and mitochondrial DNA content, and birth weight cannot be excluded.

The major strengths of this study are that we tested the different windows of exposure, and made use of two independent birth cohorts in Southern (Spain) and Western (Belgium) Europe. Also, we used new methods and their assumptions to study causal interference (Valeri and VanderWeele 2013). Nevertheless, this mediation analysis gives only estimates of the DE, IE, and TE and the method assumes no uncontrolled confounding. Furthermore, our results of the association between prenatal air pollution exposure and mtDNA content are in line with those of Janssen et al. (2012) in the same birth cohort (ENVIR*ON*AGE) with a smaller sample size. We also added more information by performing a mediation analysis that supports our hypothesis that mitochondrial damage may be a causal intermediate in biological mechanisms linking air pollution exposure to birth outcomes, which may provide some mechanistic clues to the adverse effects of early exposure to air pollution observed in humans.

In conclusion, we have shown that prenatal NO_2_ exposure was inversely associated with both placental mtDNA content and birth weight. Considering the high levels of NO_2_ in urban areas, which are increasing worldwide, this study indicates the relevance of further exploring this biological pathway linking early air pollution exposure and complications at birth.

## Supplemental Material

(398 KB) PDF
