# Supplementary material for: Prenatal Ambient Air Pollution, Placental Mitochondrial DNA Content, and Birth Weight in the INMA (Spain) and ENVIRONAGE (Belgium) Birth Cohorts
Source: Environ Health Perspect. 2015 Aug 28;124(5):659–65. doi: 10.1289/ehp.1408981 (PMC4858384; doi:10.1289/ehp.1408981)
Supplement: (398 KB) PDF [file ehp.1408981.s001.acco.pdf]

Environ Health Perspect
DOI: 10.1289/ehp.1408981

Note to Readers: *EHP* strives to ensure that all journal content is accessible to all readers. However, some figures and Supplemental Material published in *EHP* articles may not conform to 508 standards due to the complexity of the information being presented. If you need assistance accessing journal content, please contact ehp508@niehs.nih.gov. Our staff will work with you to assess and meet your accessibility needs within 3 working days.

Supplemental Material

Prenatal Ambient Air Pollution, Placental Mitochondrial DNA Content, and Birth Weight in the INMA (Spain) and ENVIR*ON*AGE (Belgium) Birth Cohorts

Diana B.P. Clemente, Maribel Casas, Nadia Vilahur, Haizea Begiristain, Mariona Bustamante, Anne-Elie Carsin, Mariana F. Fernández, Frans Fierens, Wilfried Gyselaers, Carmen Iñiguez, Bram G. Janssen, Wouter Lefebvre, Sabrina Llop, Nicolás Olea, Marie Pedersen, Nicky Pieters, Loreto Santa Marina, Ana Souto, Adonina Tardón, Charlotte Vanpoucke, Martine Vrijheid, Jordi Sunyer, and Tim S. Nawrot

Table of contents

Table S1. Data from each campaign and predictor variables of air pollution in each cohort
Figure S1. GAM models that show the relation between (A) NO_2 exposure (µg/m3) during the entire pregnancy and mtDNA content, (B) NO_2 exposure (µg/m3) during the entire pregnancy and birth weight (g), and (C) mtDNA content and birth weight (g). This model provides p-values for the null hypothesis of no linearity. 1: ENVIRONAGE, 2: INMA
Table S2. Percent change in placental mtDNA content in association with prenatal NO_2 exposure in the four different INMA subcohorts
Table S3. Association between pregnancy average NO_2 exposure and birth weight in the four different INMA
subcohorts

Table S4. Mediation analysis of the estimated effects of prenatal NO_2 exposure (µg/m3) on birth weight through placental mtDNA content in the pooled sample and the boys of the pooled sample

Table S5. Percent change in placental mtDNA content in association with prenatal NO_2 exposure in INMA mothers that spent ≥ 15 hours a day at home

Table S6. Association between prenatal NO_2 and birth weight in INMA mothers that spent ≥ 15 hours a day at home

Table S1. Data from each campaign and predictor variables of air pollution in each cohort

Cohort	No. Passive samplers	Campaign dates	Predictor variables	R^2 model	Number of monitoring stations
**INMA-Asturias**	67	June 2005<br>November 2005	Altitude<br>Distance to nearest road[a]<br>Agricultural or forest land cover (300m-buffer)	0.521	4
**INMA-Gipuzkoa**	85	February 2007<br>June 2005	Altitude (3 cat)<br>Valley factor<br>Distance to nearest road[a] (MDI[b] > 20000)<br>Urban land cover (100m-buffer)<br>Industrial land cover (300m-buffer)	0.509	3
**INMA-Sabadell**	57	April 2005<br>June 2005<br>October 2005<br>March 2006	Altitude<br>Urban or industrial land cover (500m-buffer)<br>Road type (minor, major, secondary road)	0.750	1
**INMA-Valencia**	93	April 2004<br>June 2004<br>November 2004<br>February 2005	Kriging[c]<br>Industrial or urban land cover (500m-buffer)<br>Distance to nearest major road[a] (MDI[b] > 10000)	0.730	7
**ENVIR*ON*AGE**	N/A	N/A	Kriging[d]<br>Land use[d]<br>Road emission and locations[e]<br>Point sources, characteristics and locations[e]	0.820	64

[a]Distance to the nearest major road (in logarithms)
[b]MDI: Mean daily traffic count
[c]Mean of estimated NO_2 from kriging among campains
[d]Predictor variables of the land-use regression model for the background (RIO)
[e]Predictor variables of the emission for the bi-gaussian plume model.

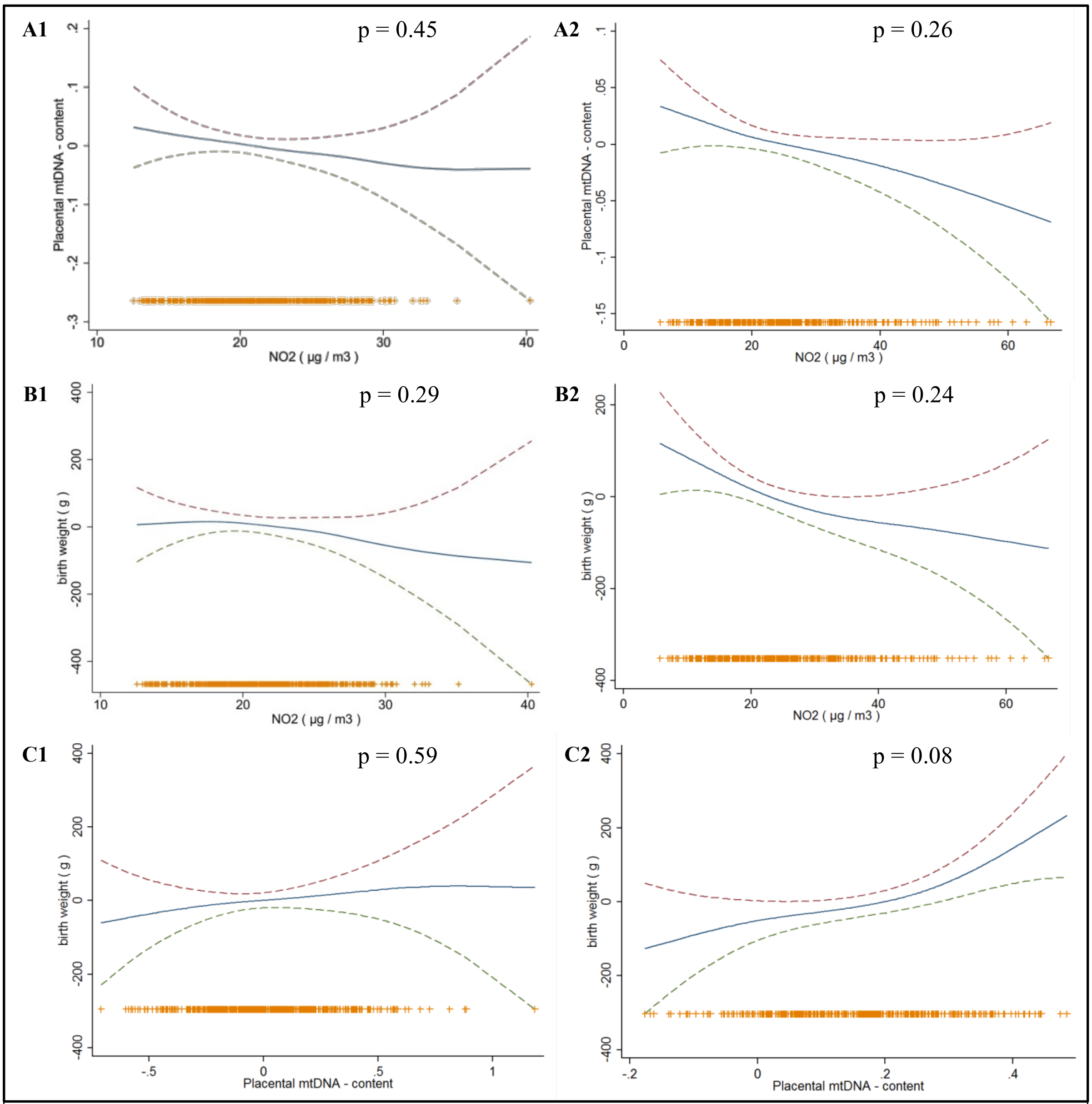

Figure S1. GAM models that show the relation between (A) NO_2 exposure (µg/m3) during the entire pregnancy and mtDNA content, (B) NO_2 exposure (µg/m3) during the entire pregnancy and birth weight (g), and (C) mtDNA content and birth weight (g). This model provides p-values for the null hypothesis of no linearity. 1: ENVIRONAGE, 2: INMA

Table S2. Percent change in placental mtDNA content in association with prenatal NO_2 exposure in the four different INMA subcohorts

**Pregnancy period**	**Differences in placental mtDNA content (%)**	**95% CI**	**P-value**
**Asturias (n=37)**			
Trimester 1	-13.6	-31.4, 8.8	0.20
Trimester 2	-12.8	-26.0, 2.9	0.10
Trimester 3	-16.8	-30.8,-0.07	0.05
Entire pregnancy	-15.1	-29.9, 2.9	0.09
**Gipuzkoa (n=156)**			
Trimester 1	-6.3	-12.6, 0.5	0.07
Trimester 2	-6.5	-13.0, 0.4	0.06
Trimester 3	-5.4	-12.3, 2.1	0.15
Entire pregnancy	-4.6	-11.8, 3.2	0.23
**Sabadell (n=120)**			
Trimester 1	-2.6	-7.3, 2.3	0.29
Trimester 2	-3.4	-8.3, 1.8	0.16
Trimester 3	-3.4	-8.3, 1.8	0.19
Entire pregnancy	-4.1	-9.4, 1.6	0.15
**Valencia (n=63)**			
Trimester 1	-3.1	-8.5, 2.6	0.27
Trimester 2	-1.6	-8.1, 2.6	0.27
Trimester 3	-2.6	-8.3, 3.4	0.38
Entire pregnancy	-2.8	-8.9, 3.7	0.38

Effect size was estimated for each 10 $\mu g/m^3$ increment in exposure to NO_2 at each mother's residence during the corresponding period;

Models were adjusted for newborn's sex, maternal age, maternal smoking status, gestational age (linear and quadratic), pre-pregnancy BMI, parity, ethnicity, season of birth, and education;

Table S3. Association between pregnancy average NO_2 exposure and birth weight in the four different INMA subcohorts.

INMA subcohort	N	Differences in birth weight (g)	95% CI	*p*-value
**Asturias**	37	-155.6	-378.3, 67.1	0.16
**Gipuzkoa**	156	-75.6	-168.1, 13.1	0.08
**Sabadell**	120	-110.3	-178.3, -42.4	0.002
**Valencia**	63	-11.8	-20.1, 12.4	0.83

Effect size was estimated for each 10µg/m^3 increment in pregnancy average exposure to NO_2 at each mother's residence during the corresponding period.

Models were adjusted for newborn's sex, maternal age, maternal smoking status, gestational age (linear and quadratic), pre-pregnancy BMI, parity, ethnicity, season of birth, and education

Table S4. Mediation analysis of the estimated effects of prenatal NO_2 exposure (µg/m3) on birth weight through placental mtDNA content in the pooled sample and the boys of the pooled sample.

Exposure period	Proportion of mediation (%)	95% CI	*p*-value
**Pooled data (n = 926)**			
Trimester 1	1.4	-2.6, 5.5	0.54
Trimester 2	2.9	-2.6, 7.9	0.29
Trimester 3	3.2	-2.2, 8.1	0.24
Entire pregnancy	2.2	-2, 6.1	0.29
**Boys of pooled data (n = 471)**			
Trimester 1	1.5	-4.7, 7.0	0.62
Trimester 2	4.3	-0.2, 8.5	0.06
Trimester 3	6.4	2.4, 10.1	0.002
Entire pregnancy	2.8	-0.8, 6.2	0.12

Effect size was estimated for each 10µg/m^3 increment in exposure to NO_2 at each mother's residence during the corresponding period.

Models were adjusted for newborn's sex, maternal age, maternal smoking status, gestational age (linear and quadratic), pre-pregnancy BMI, parity, ethnicity, season of birth, and education.

Table S5. Percent change in placental mtDNA content in association with prenatal NO_2 exposure in INMA mothers that spent ≥ 15 hours a day at home.

Exposure period	N	Differences in placental mtDNA content (%)	95% CI	P-value
**Trimester 1**	214	-6.1	-9.5, -2.5	<0.01
**Trimester 2**	214	-7.5	-11.1, 3.8	<0.01
**Trimester 3**	214	-7.8	-11.4, -4.0	<0.01
**Entire pregnancy**	214	-8.9	-12.8, -4.8	<0.01

Effect size was estimated for each 10 $\mu g/m^3$ increment in exposure to NO_2 at each mother's residence during the corresponding period;
Models were adjusted for newborn's sex, maternal age, maternal smoking status, gestational age (linear and quadratic), pre-pregnancy BMI, parity, ethnicity, season of birth, and education;
Results followed the same direction in all 4 INMA subcohorts (data not shown);
INMA subcohort was included as random effect.

Table S6. Association between prenatal NO_2 and birth weight in INMA mothers that spent ≥ 15 hours a day at home.

Exposure period	N	Differences in birth weight (g)	95% CI	P-value
**Trimester 1**	214	-64.9	-114.1, -15.6	0.01
**Trimester 2**	214	-65.9	-118.5, -13.3	0.01
**Trimester 3**	214	-75.1	-127.9, -22.2	< 0.01
**Entire pregnancy**	214	-83.7	-141.8, -25.6	< 0.01

Effect size was estimated for each 10µg/m^3 increment in exposure to NO_2 at each mother's residence during the corresponding period.
Models were adjusted for newborn's sex, maternal age, maternal smoking status, gestational age (linear and quadratic), pre-pregnancy BMI, parity, ethnicity, season of birth, and education;
Results followed the same direction in all 4 INMA subcohorts (data not shown);
INMA subcohort was included as random effect.
